# Effects of digital transformation on firm performance: The role of IT capabilities and digital orientation

**DOI:** 10.1016/j.heliyon.2024.e27725

**Published:** 2024-03-08

**Authors:** Virginia Barba-Sánchez, Angel Meseguer-Martínez, Ricardo Gouveia-Rodrigues, Mario L. Raposo

**Affiliations:** a Business Administration Department, ESII, Universidad de Castilla-La Mancha, Albacete, Spain; b NECE-UBI – Research Centre for Business Sciences, Universidade da Beira Interior, Covilhã, Portugal

**Keywords:** IT capability, Sociotechnical systems approach, Digital transformation, Digital orientation, Firm performance

## Abstract

Organisations undertake profound changes to fit in a rapidly evolving digital setting. However, although the IT capabilities of the organisational members play a critical role in this, the mechanism driving IT capabilities towards enhanced firm performance is not fully understood. A theoretical model to analyse the role of digital orientation and digital transformation in this relationship is introduced and tested on a set of 246 firms through the Partial Least Squares-Structural Equation Modeling method (PLS-SEM). This research contributes to the literature by introducing the social aspect to the study of technology management, delving also into the antecedents of digital transformation. Results confirm a positive effect of IT capabilities on firm performance through the development of a digital orientation and the digital transformation of the organisation.

## 1 Introduction

Digitization has revolutionised human behaviour. In the last years, we have witnessed a shift in social interactions to the digital dimension. This changing environment inevitably exerts a deep impact on organisations that must face threats and opportunities from this technology-driven revolution [1,2]. Hence, organisations undertake profound changes in their structures, processes, and offerings to not only survive but thrive in this new setting. In line with this, research consistently indicates that IT capabilities positively affect firm performance [3,4]. However, the efforts to explain the mechanism that drives such capabilities towards enhanced performance have yielded inconclusive results [1,5,6]. Therefore, these relevant topics related to the organisational management of technology are yet unknown [7].

In accordance with this, the digital transformation has revolutionised businesses [8], as shown by its importance for the industry as highlighted in management surveys [9]. Consequently, digital transformation has garnered substantial research interest. This increasingly studied concept is recognised as a pivotal factor for competitiveness. Research, commonly associates this concept with firm investments in IT, consistently affirms the positive effect of digital transformation on firm performance [10,11]. This effect is particularly emphasized as a catalyst in the relation between IT capabilities and firm performance [6,12].

Nevertheless, not all existing research agrees on the positive effect of digital transformation on firm outcomes [1,13], and further research is needed to understand better how digital transformation operates [2,14].

The organisation's members are the holders of the IT capabilities, which enable the management of organisational IT resources and the individual alignment toward digital solutions for daily tasks. Cavalcanti et al. [7, p.3] define individual adoption of digital transformation “as the degree to which disruptive and transformative technologies are adopted and/or accepted by individuals, whether employees, consumers, customers or citizens, after an improvement event or development of a new product, process or innovation”. At the organisational level, this is reflected in adapting the structures and strategies to harness the opportunities that digital technologies bring [15].

In this respect, the digital orientation of the organisation, as a social construct which reflects the philosophy of the organisation to set the foundations of their activities on the potentialities of digital technologies [15], assumes a vital role in the effective steering of the organisational IT capabilities towards an effective digital transformation and, consequently, improved performance. This concept captures how the capabilities, values and beliefs of organizational members shape the firm's philosophy, ultimately leading to improved performance [16]. It involves the creation or adaptation of digital capabilities, coordination of digital ecosystems, and reshaping of digital architectures [15]. As a strategic orientation, evidence shows a positive effect on performance. However, as Cavalcanti et al. [7] conclude, further research is needed to understand how digital orientation leads to performance gains.

Therefore, the social aspects are critical in the study of technology management [17,18]; yet these effects have been poorly addressed. Although extant research tends to show positive effects of IT capabilities on performance [4], externalities arising from these capabilities can hinder the relation [19,20]. In this sense, digital transformation has been considered as an explanatory factor [6], however subject to controversy [1,13].

We consider that the limited focus on the interaction between social and technical factors in the analysis of the effects of IT capabilities on organisations presents a challenge to fully comprehend how these capabilities help organisations in aspects as important as a successful digital transformation or the improvement of organisational results. Therefore, we adopt a novel focus, introducing the digital orientation as a concept of social nature to the study of the links between IT capabilities and firm performance, and the role of digital transformation to explain the mechanisms that enable firms to harness the benefits of IT capabilities.

Based on this, we pose the following research questions:

RQ1 What is the impact of IT Capabilities on the Organisation's Performance?

RQ2 What role does Digital Orientation and Digital Transformation play in the association between IT Capabilities and Firm Performance?

In this study, we set off to introduce a theoretical framework to explain how IT capabilities lead to enhanced firm performance, with a focus on the mediating roles of digital orientation and digital transformation in this association. This model is subsequently validated empirically using a dataset from Spanish firms.

We contribute to the literature on IT capabilities and address the discussion on the mechanisms that explain its relationship with organisational performance. To this end, we employ a theoretical approach to analyse the importance of social aspects in technology management. Additionally, we contribute to the study of the antecedents of digital transformation, also considering the importance of the interactions among social and technical factors crucial for technology-related changes in organisations.

The remainder of the study is structured as follows: the second section reviews the literature on the relevant concepts and discuss their relationships, the third section describes the methods, the fourth presents the results, and, in the final section, the results are discussed, and conclusions are presented.

## 2 Theory

### 2.1 IT capability and firm performance

IT capability refers to the ability to acquire, deploy, combine, and reconfigure IT resources to support and enhance business strategies and work processes [21,22]. This capacity deals with the organisational ability to enhance effectiveness in information management to improve its competitive position [23].

This concept has been subject to extensive research for over two decades. During this period, authors such as Bharadwaj [3] and Dale Stoel and Muhanna [5] have emphasized its complexity. The capability involves the adequate combination of IT-related resources, skills, and knowledge with resources and activities of different nature to yield desired outcomes [2]. Consequently, research has highlighted that mere investments in IT are insufficient for firms to achieve their objectives [5]. These investments must translate into the development of IT capacities within the organisation to effectively manage IT resources and technologies [24]. This includes artificial intelligence, big data analytics, and social platforms, to generate positive improvements for firms [2].

When successfully developed, IT capability yields remarkable benefits for firms in the form of enhancements of organisational agility, supply chain management, innovation, and the transmission of information essential for synchronous decision making, and collaboration -both internal and external- [19,[25], [26], [27], [28]]. In addition, in the context of economic crises and highly competitive environments, the beneficial impact of IT on business performance appears to be even more pronounced [29,30].

A specific line of research focuses on the relation between IT capability and firm performance [[3], [4], [5], [6],^26^,^31^]. Research in this line, regards IT capability as an enhancer of firm performance [3,4,6,26].

Scholars overwhelmingly agree that the IT capability of firms enhances their performance [3,5,31]. This consensus arises from the understanding that IT capability allows firms to effectively mobilise, deploy, and leverage IT resources, in conjunction with other organisational resources and capabilities [6]. Consequently, the availability of IT capability leads to improvements in firm performance. Based on this, we propose our first hypothesis:

H1 The IT capability of firms influences their performance.

### 2.2 The mediating effects of digital orientation

Despite the common agreement on the positive effect on one another, the relation between the IT capability of firms and their performance is more complex than assumed in the early stages of research [5]. Consequently, the link is not yet clear [4,6,31]. Research has questioned the direct effect and has, accordingly, directed attention towards additional factors to help explain not so much if but how the IT capabilities enhance firm performance. Findings suggest that the relationship depends to a large extent on environmental conditions [5,31], the types and nature of the IT capability [5], and organisational factors such as the innovation climate [4]. Additionally, digital transformation has emerged as a key mediator in the relation between IT capability and firm performance, as evidenced by empirical work on the antecedents of digital transformation of [6].

Given that the IT capability is grounded in a specific set of resources, the main theoretical background for the contributions in this line of research is -not surprisingly-the resource-based view (RBV) [3]. This is because it attributes superior financial performance to organisational resources and capabilities, and the IT capability primarly aims to harness the potential of IT resources [19] in the pursuit of competitive advantage [23]. Yet, the interaction between the human factor and the digital resources, upon which the successful application of the IT capabilities relies [32], has been overlooked, with exemptions such as the application of sociomaterialism to the conceptual development of the IT capability by Kim et al. [33].

Organisational IT capabilities are closely linked with the reservoir of human skills and abilities necessary to adequately utilise the resources and assets available [4]. In this respect, approaches such as the sociotechnical systems approach (STS) offer a framework for analysing organisational technology-related change [34]. This approach views the organisation as a system with two interrelated subsystems, the technical and the social. The social subsystem encompasses the attributes of individuals, such as attitudes, skills, and values, along with the relations among them [35]. The underlying assumption is that organisations use technology to gain a competitive advantage and remain viable [34], stressing the importance of social and technical factors and their interactions [18].

Similarly, digital orientation can be defined as the propensity of organisations to draw on digital technologies to manage their structures and activities efficiently. It is a strategic orientation [15], reflecting the organisational beliefs on how to conduct their activities [36]. The group of individuals of digitally oriented organisations -i.e. the social subsystem-is prone to draw on digital technologies to orchestrate activities. This reflects a philosophy which drives organisations to rely on a deeply rooted set of values and beliefs to perform activities with enhanced performance [16]. Hence, the concept captures how organisations manage their capabilities to reap the potentialities of digital technologies, and how, in turn, they can create and coordinate digital ecosystems and reconfigure their digital architectures [15].

Given that the digital orientation reflects the extent to which an organisation is prone to use digital technologies, which entail immense potentialities for organisations able to harness their complexity, the digital orientation of organisations can be an explaining factor for the relation between IT capabilities and organisational performance.

The complexity of digital technologies [5] and their profound impact on markets and competition call for improved managerial and organisational alignment [37]. At the individual level, the availability of IT capabilities enables individuals to reap the potential of digital technologies. Additionally, at the organisational level, the availability of IT capabilities within the social subsystem leads to the necessary managerial and organisational alignment to use digital technologies for their activities. This creates, a climate favourable to such technologies. IT capabilities thus facilitate the coordination of the human side of the organisation with factors and resources of technological nature [38], generating a digital orientation.

Digitally oriented organisations are generally agreed to have improved performance. For instance, Kindermann et al. [15] provide empirical evidence for the positive relation between digital orientation and firm performance. However, organisations may have to incur costs to expand their business models based on the digital orientation, which may ultimately not compensate for the benefits. Nevertheless, digitally oriented organisations are in a better position in terms of innovation, customer satisfaction, returns and overall performance [38]. Hence, the IT capability is an underlying condition for the organisation's digital orientation, which, in turn, leads to enhanced performance. Based on this, we introduce our second hypothesis:

H2 The digital orientation mediates the relation between IT capability and organisational performance.

### 2.3 The mediating effects of digital transformation

As previously mentioned, the digital orientation steers organisations towards using digital technologies, paving the way for the organisation's digital transformation. Digital transformation is an evolutionary process that leverages digital capabilities and technologies to create value for business models, operational processes, and customer experiences [39]. It transcends specific technological changes and is transformational [40] since it usually leads to the development of new business models [2]. Digital transformation is characterised by shifts towards big data, artificial intelligence, analytics, cloud, mobile and social media platforms [6]. It results in new value propositions which usually co-exist with the traditional ones [41], introducing profound changes in the distribution channels, sources of innovation, business practices, stakeholders relations, and, consequently, in the creation of value [8].

Reis and Melão [2] conducted a rigorous meta-review of the existing literature, identifying six dimensions related to digital transformation: business models, digital business, technologies, sustainability, human resources, and smart cities. However, organizational, technological, and social dimensions remain the primary ones. In this sense, Costa Melo et al. [1] highlight the implications of digital transformation for the strategy and innovation of the business model in the context of industry ecosystems, composed of suppliers, competitors, customers, etc.

Digitally oriented organisations introduce in an effective manner new organisational structures and responsibilities, enabling them to reap the potentialities of technological change, as well as to apply digitization. This, in turn, facilitates the organisation's digital transformation [15]. Therefore, the availability of IT capabilities within the organisation is necessary for achieving digital transformation. However, organisations must also be digitally oriented to effectively direct their IT capabilities toward digital transformation. Based on this, we pose our third hypothesis:

H3 The digital orientation mediates the relation between IT capability and digital transformation.

Research has traditionally drawn on digital transformation to explain the relation between IT capability and results as it positively affects this relation [6,42]. Firms with superior IT capabilities can instigate digital transformation by redesigning and rethinking existing business processes and transforming traditional products, services, and customer offerings into digital offerings [6]. Additionally, digital transformation helps firms in enhancing their performance as it enables them to increase the degree of customisation, customer satisfaction and decreased costs, thereby improving overall customer offerings [10,43]. Thus, organisations with digitally embedded business processes obtain enhanced performance benefits from their IT capabilities [6].

However, the social aspect of technology management also plays a vital role in digital transformation and firm performance. Firms endowed with IT capabilities are inclined to develop a digital orientation. Subsequently, the digital orientation -as a strategic orientation of firms-caters to changes induced by digital technology [15], driving the organisation towards a digital transformation, which leads to improved performance.

We posit that the relation between the IT capabilities and FP is explained by the firm's ability to generate a digital orientation based on its IT capabilities. Subsequently, leveraging this strategic orientation, the firm effectively achieves digital transformation, contributing to enhanced performance. Thus, we introduce our fourth hypothesis:

H4 The relationship between the IT capabilities and firm performance is doubly mediated, first by digital orientation and then by digital transformation.

**Fig. 1:**
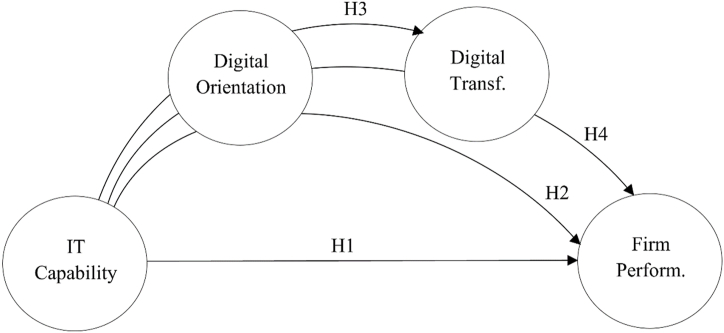
Theoretical model.

In summary, Fig. 1 illustrates the set of relationships hypothesised in the proposed research model.

## 3 Material and methods

### 3.1 Data collection, sample, and analysis techniques

To examine the relationships between ICT capacity, digital orientation and digital transformation, as well as the relations between the latter and the firm performance, we gathered data through online questionnaires from a sample of firms from the Analysis System of Iberian Balances (SABI) database. The SABI database contains detailed information of over 2,900,000 Spanish and more than 900,000 Portuguese companies. In our case, our study population focuses on Spanish companies in general, so the SABI database is a valuable resource. The contact persons at SABI, who serve as legal representatives of the company, such as Chief Executive Officers (CEOs) or owners, received an invitation to participate by email with the link to the form. To adhere to ethical guidelines for questionnaire surveys, an informed consent document was developed. The document includes the aspects required to provide the participants with the necessary information about the research and it complies with the regulations in force regarding personal data protection. The Social Research Ethics Committee (SREC) of the authors’ university has verified that this study adheres to ethical standards established for social research (CEIS-736254-X1Q3).

Before the launch, the required minimum sample size was calculated to confirm the validity of our model [44]. For this purpose, we chose the inverse square root method [45] because it is conservative and overestimates the sample size required to render an effect significant at a given power level. In our case, the minimum path coefficient ranges from 0.11 to 0.20, so the minimum sample size is 155 observations to render the corresponding effect significant at 5%. To achieve the minimum sample size, invitations to complete the online survey were sent to 1550 companies. Finally, 246 questionnaires were obtained during the 20 days that the survey was open on the Google Forms platform (from 8 to 28 April 2022), resulting in a response rate of 15.87%. The results show that our sample size (246) exceeds the minimum established in the inverse square root method.

With regards to the sample, it is composed of diverse-sized firms with a prevalence of micro-SMEs (less than 10 employees), as more than 70% have fewer than 10 employees (see Table 1). This distribution is in line with the population. However, micro-SMEs are somewhat underrepresented in the sample (as observed in Table 1) surely due to a lower response rate from this category of firms.

**Table 1:** Firm size.

Number of employees	Sample		Population (SABI)	
	Count	Percent	Count	Percent
**Without employees**	48	19.5122	306,397	26.5049
**1–9**	126	51.2195	622,166	53.8205
**10–49**	61	24.7967	191,780	16.5899
**50–249**	8	3.2521	30,082	2.6022
**≥250 employees**	3	1.2195	5577	0.4824
**Total**	246	100.0000	1,156,002	100.0000

a The firm population in SABI corresponds to those Spanish companies that filled in the information on the number of employees.

Structural Equation Modelling (SEM) is applied using the partial least square (PLS) SEM-variance analysis. This methodology is particularly recommended to test mediation hypotheses [46], as is the case here. Specifically, we used the Smart PLS 3.3.9 application [47].

### 3.2 Measures

*IT capability* (ITC). We drew on the scale of Lu and Ramamurthy [19], which consists of 12 items related to IT infrastructure and business-spanning capability. For each item, respondents were asked to evaluate their organisation's IT relative to other firms in their industry on a 1–5 scale (1 = poorer than most, 5 = superior to most). This scale is widely accepted [6].

*Digital orientation* (DO). The scale of Khin and Ho [38] was used to measure this variable. The scale draws on four items related to personal intention and commitment to using digital technologies. For each item, respondents indicated their agreement or disagreement with a series of statements, where one represented "strongly disagree" and five "strongly agree".

*Digital transformation* (DT). We used the scale of Singh et al. [17] which comprises three items addressing their organisation's digital transformation level. Responses were provided on a 5-point scale (1 = strongly disagree, 5 = strongly agree).

*Firm's performance* (FP). We used the scale of Lee et al. [48], which comprises seven items addressing their organisations' financial and non-financial results. A 5-point scale measured all items (1 = strongly disagree, 5 = strongly agree).

## 4 Results

Before delving into the structural model, the reliability and validity of the measurement model were tested. The individual reliability of each item was assessed and as all items had factor loadings surpassing the 0.707 threshold, none were excluded. Next, we assessed construct reliability through the Cronbach's Alpha, Dijkstra Henseler's rho_A, and the Composite Reliability. All of them showed values above 0.7 (Table 2), confirming their reliability. Then, we verified the convergent validity of the constructs through the Average Variance Extracted (AVE), with all values above the 0.5 benchmark (Table 2). Finally, the Heterotrait-Monotrait (HTMT) and Fornell-Lacker criteria confirmed the discriminant validity of the constructs (Table 3).

**Table 2:** Reliability estimates and convergent validity of the measurement model.

Construct^a^	Cronbach's Alpha	Dijkstrqa-Henseler's rho_A	Composite reliability (CR)	Average variance extracted (AVE)
Digital Orientation	.921	.922	.944	.808
Digital Transformation	.945	.947	.964	.900
IT Capacity	.956	.961	.961	.674
Firm Performance	.920	.927	.935	.675

a All constructs are estimated in Mode A.

**Table 3:** Discriminant validity^a^ of the measurement model based on Fornell-Larcker and HTMT_0.90_ Criteria.

Construct	DO	DT	ITC	FP
Digital Orientation (DO)	**.899**	*.696*	*.889*	*.649*
Digital Transformation (DT)	.651	**.949**	*.600*	*.630*
IT Capacity (ITC)	.844	.582	**.821**	*.626*
Firm Performance (FP)	.609	.593	.599	**.821**

a Diagonal elements (bold) represent the square root of the variance shared between the constructs and their measures (AVE). Italic values above the diagonal elements are HTMT_0.90_ values. Values below the diagonal elements are the correlations between constructs.

To evaluate the structural model, the VIF values were used to check for collinearity problems among the constructs. All the values remained below 5, which is the maximum established in the literature [49]. Then, the goodness of fit was confirmed through the Standardised Root Mean Square Residual (SRMR), with values consistently below 0.08 [50]. Furthermore, a bootstrapping process (10,000 subsamples) based on the confidence interval percentiles was employed to verify the significance of the path coefficients [51].

As observed in Table 4, the results indicate that ITC influences FP significantly (H1: β = 0.260; p < .01). Hypothesis 1, hence, is accepted. Additionally, the R^2^ values of DT and FP are above 0.33, and the R^2^ values of DO are above 0.67. Consequently, the explanatory power of the model is moderate but tends to be substantial [52]. With regards to the individual contributions of the constructs, DT makes the largest contribution to FP (.195). However, DT is contingent on DO (.364). This is confirmed when the extent to which DO contributes to explaining the R^2^ DT is calculated (f^2^ = 0.153).

**Table 4:** Direct effects on endogenous constructs.

Construct	Direct Effect^a^	*t*-Value^b^	*p* Value^b^	PCI^b^	Explained Variance (R^2^)	*f*^*2*^
**Firms Performance (R**^2^**= .457)**						
DT	.328	4.257	.000	−0.171, .471]	.195	.114
DO	.175	1.805	.071	[-.026, .359]	.107	.014
H1: ITC	.260	2.891	.004	[.087, .439]	.155	.035
**Digital Transformation (R**^2^**= .426)**						
DO	.560	5.401	.000	[.350, .756]	.364	.153
ITC	.107	.977	.329	[-.103, .321]	.062	.006
**Digital Orientation (R**^2^**= .719)**						
ITC	.848	41.967	.000	[.804, .883]	.719	2.564

EC: Endogenous construct; CV: Control Variable; PCI: Percentile Confidence Interval.

a Paths from hypothesis assessed by applying a two-tailed test at a 5% of significance level [2.5%, 97.5%].

b Bootstrapping based n = 10,000 bootstrap samples.

Regarding the mediating effects, the total effects of the ITC on FP and DT are shown in Table 5. All total effects surpass the direct effects, implying the existence of mediation or indirect effects [53]. Thus, according to Nitzl et al. (2016), we confirm significant partial mediation relationships between ITC and FP through DO (H2: β = 0.149; p < .1) and through DO and DT (H4: β = 0.155; p < .01). The assessment of the indirect effects of ITC on DT through DO (H3: β = 0.475; p < .001) confirms a total mediation, given the non-significance of the direct effect (β = 0.107; p > .1) mentioned earlier. In addition, these results are confirmed by the size of the indirect effects calculated with the Variance Accounted For index (VAF), for which -following Hair et al. (2014)- values between 20% and 80% represent partial mediation, while values above 80% total mediation.

**Table 5:** Summary of mediating effect tests.

Hypothesis	Total effect Path (*p-*Value)^a^	Direct effect Path (*p-*Value)^a^	Indirect effect		
			Path (*p-*Value)^a^	PCI^b^	VAF (%)
H2: ITC → DO → FP	.599 (.000)	.260 (.000)	.149 (.070)	[-.021, .303]	24.87
ITC → DT → FP			.035 (.333)	[-.031, .115]	5.84
H4: ITC → DO → DT→ FP			.155 (.001)	[.079, .263]	25.87
H3: ITC → DO → DT	.582 (.000)	.107 (.329)	.475 (.000)	[.299, .646]	81.61
DO → DT→ FP	.359 (.000)	.175 (.071)	.184 (.001)	[.093, .306]	51.25

PCI: Percentile Confidence Interval.

a Paths from hypothesis assessed by applying a two-tailed test at 5% of significance level [2.5%, 97.5%].

b Bootstrapping based n = 10,000 bootstrap samples.

**Fig. 2:**
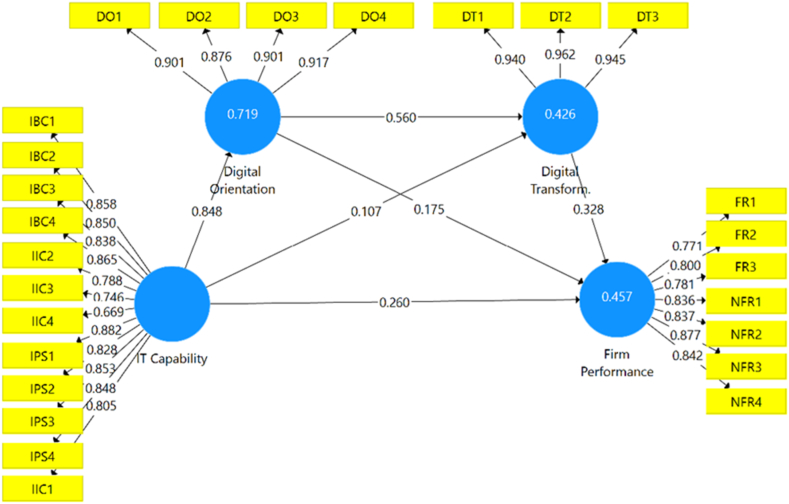
PLS Estimation of the model.

In summary, the first and third hypotheses proposed in our theoretical model were empirically supported**,** whereas H2 and H4 were partially supported. Fig. 2 summarises the standardised regression coefficients and the proportions of the explained variance (R^2^).

## 5 Discussion and conclusion

In this study, we introduce social factors to delve into the dynamics of IT capabilities and the mechanism that drives such capabilities toward enhanced organisational performance. We also contribute to the literature on digital transformation, focusing on its antecedents. The theoretical approach applied poses a novel focus in a body of literature dominated by the RBV perspective, allowing us to highlight the necessary social-technological interaction for effective digital transformation.

Based on this, we introduce a theoretical framework to explain whether the IT capabilities lead to organisational performance. Additionally, our exploration encompasses the mediating role of the digital orientation and digital transformation in this relation.

Drawing on the concept of digital orientation allows us to (1) delve into the antecedents of digital transformation, an under-researched domain [6,15], and (2) introduce a social approach into the study of technology management a social factor aims to explain how firms can effectively steer their IT capabilities towards digital transformation, ultimately leading to increased performance. Thus, we expand the current focus on technological resources of most of the existing literature rooted on RBV. Instead, we broaden the scope to encompass both the social and technological resources needed to manage technology effectively [18]. This shift emphasises the importance of the interplay between technological resources, human skills, know-how and the organisational routines at the organisational level in a way that competitors cannot easily imitate and that, therefore, provides a source of competitive advantage [54].

The empirical test allowed us to validate the theoretical model, and further conclusions can be drawn from it. First, we offer additional support to the positive relation between IT capabilities and firm performance -in line with the literature [[3], [4], [5]]-. This provides a positive answer to our first research question.

Then, the positive relationship between the digital orientation of the firm and its performance has been confirmed following extant research [15] as well as that of digital transformation and organisational performance [6,10]. Additionally, digital orientation -as a strategic factor of the organisation contingent on its members-proves to be an important antecedent of digital transformation. Both digital orientation and digital transformation drive the effects of the IT capabilities to enhance organisational performance.

With its distinctive features, technology management presents a complex task with great potential for the firm [15]. Such a task requires individual and organisational skills, routines, and architectures that allow the firm to reap the potential of technology. The results of this study unveil a socially rooted mechanism that drives the IT capabilities towards improved performance. Firms can steer their IT capabilities through a digital orientation towards an effective digital transformation, yielding improved results. Based on this, we also answer our second research question.

### 5.1 Implications

The current research contributes to our understanding of the relevance of the social aspects on technology management, in line with demands of [18]. In particular, at the organizational level, as suggested by Arias-Pérez et al. [55]. Technology management is complicated, yet critical to cope with environmental conditions. It depends on the organisation's members as the holders of the IT capabilities and the firm capacity to harness the potential benefits of these. Introducing a variable of social nature like digital orientation sheds light on the mechanisms driving IT capabilities towards digital transformation and enhanced firm performance. It underscores the need for an effective imbrication of social and technological factors for a successful technological management which needs to be further explored.

The study bears practical implications. It seeks to support practitioners in making sensible strategic decisions concerning the implementation of digital change to respond to evolving digital technologies. We provide managers with insights into the digital orientation of their organisations as a critical domain worthy of consideration to benefit from investments in IT and the digital orientation effectively.

The mere investment in digital technologies, even with the presence of IT capabilities within the organisation, falls short of achieving digital transformation and its desired effects. The organisation's digital orientation is needed to achieve a digital transformation and subsequently improve performance. As a strategic orientation for the firm, the digital orientation calls for a rearrangement of the organisational processes and structures, signifying a strategic shift [15].

### 5.2 Limitations and further research

Our study is not without limitations. Studies such as those of Chen et al., and Dale Stoel and Muhanna [5,31] show that environmental variables affect the relations among the variables considered. Additionally, this study does not consider the effect of managerial involvement on the attitude towards change. The scope of the study did not allow us to encompass these aspects. However, we believe they are a relevant venue for future research. The sample is restricted to Spanish firms, and future research may consider an international sample or samples from other countries to compare results and identify potential regional biases. In this vein, the consideration of further variables like IT investment could facilitate multigroup analyses, offering further insights. Finally, the current study draws on a transversal analysis. Longitudinal studies can be of interest, especially considering the dynamic nature of the digital transformation [56]. This can help shed light on the capacity of digital transformation to reconfigure the firm's resources and structures, with which the relations studied may evolve significantly over time.

## Ethics statement

This study was reviewed and approved by The Social Research Ethics Committee (SREC) of the authors’ university, with the approval number: CEIS-736254-X1Q3.

## Funding

This work was supported by:

- University of Castilla-La Mancha (UCLM), Spain, and the 10.13039/501100008530European Regional Development Fund (ERDF) under Grant 2022-GRIN-34373.
- NECE-10.13039/100007691UBI, Research Centre for Business Sciences, and FCT – 10.13039/501100001871Fundação para a Ciência e a Tecnologia, under Grant UIDB/04630/2020 and 10.13039/100000201DOI identifier 10.54499/UIDP/04630/2020.

## Data availability statement

The data that support the findings of this study are available from the corresponding author upon request.

## CRediT authorship contribution statement

**Virginia Barba-Sánchez:** Writing – review & editing, Writing – original draft, Visualization, Validation, Supervision, Project administration, Methodology, Investigation, Funding acquisition, Formal analysis, Data curation, Conceptualization. **Angel Meseguer-Martínez:** Writing – review & editing, Writing – original draft, Investigation, Data curation, Conceptualization. **Ricardo Gouveia-Rodrigues:** Writing – review & editing, Writing – original draft, Project administration, Conceptualization. **Mario L. Raposo:** Writing – review & editing, Project administration, Conceptualization.

## Declaration of competing interest

The authors declare that they have no known competing financial interests or personal relationships that could have appeared to influence the work reported in this paper.
