# Supplementary material for: Effects of digital transformation on firm performance: The role of IT capabilities and digital orientation
Source: Heliyon. 2024 Mar 8;10(6):e27725. doi: 10.1016/j.heliyon.2024.e27725 (PMC10951599; doi:10.1016/j.heliyon.2024.e27725)
Supplement: Multimedia component 1 [file mmc1.pdf]

Questionnaire items

IT capabilities

- IIC1: Data management services & architectures (e.g., databases, data warehousing, data availability, storage, accessibility, sharing etc.)
- IIC2: Network communication services (e.g., connectivity, reliability, availability, LAN, WAN, etc.)
- IIC3: Application portfolio & services (e.g., ERP, ASP, reusable software modules/components, emerging technologies, etc.)
- IIC4: IT facilities' operations/services (e.g., servers, large-scale processors, performance monitors, etc.)
- IBC1: Developing a clear vision regarding how IT contributes to business value.
- IBC2: Integrating business strategic planning and IT planning.
- IBC3: Enabling functional area and general management's ability to understand the value of IT investments.
- IBC4: Establishing an effective and flexible IT planning process and developing a robust IT plan.
- IPS1: We constantly keep current with new information technology innovations.
- IPS2: We are capable of and continue to experiment with new IT as necessary
  IPS3: We have a climate that is supportive of trying out new ways of using IT.
- IPS4: We constantly seek new ways to enhance the effectiveness of IT use.

Digital orientation

- DO1: We are committed to using digital technologies in developing our new solutions.
- DO2: Our solutions have superior digital technology.
- DO3: New digital technology is readily accepted in our organization.
- DO4: We always look out for opportunities to use digital technology in our innovation.

Digital transformation

- DT1: The new business processes are built on technologies such as big data, analytics, cloud, mobile and social media platforms.
- DT2: Digital technologies such as social media, big data, analytics, cloud and mobile technologies are integrated to drive change.
- DT3: The business operations are shifting toward making use of digital technologies such as big data, analytics, cloud, mobile and social media platforms.

Business results

- NFR1: Increased customer satisfaction
- NFR2: Enhanced Brand image
- NFR3: Improved Brand value
- NFR4: Increased staff productivity
- FR1: Increased turnover
- FR2: Increased market share
- Impact of the pandemics on your business
- To what extent did ICTs help the business?

Other

- Percentage of women on staff.
- Did the company carry out digital transformation projects?
- In case the answer is positive? How many?
- Number of hours devoted to ICT training during the last year.
- Does the organisation have ICT specialists on staff?
- Optional: if you wish, please state the biggest pros and cons of digital transformation for your business from your point of view.

Original questionnaire in Spanish: https://forms.office.com/r/S00q3Ph9TL
