# Supplementary material for: Effects of digital transformation on firm performance: The role of IT capabilities and digital orientation
Source: Heliyon. 2024 Mar 8;10(6):e27725. doi: 10.1016/j.heliyon.2024.e27725 (PMC10951599; doi:10.1016/j.heliyon.2024.e27725)
Supplement: Multimedia component 2 [file mmc2.pdf]

The Social Research Ethics Committee (SREC) of the University of Castilla-La Mancha has carried out the evaluation of the project "Effects of digital transformation on firm performance: The role of IT capabilities and digital orientation" in session of 23 October 2023. The SREC has verified that the project was carried out in compliance with the ethical standards developed for social research. Specifically, in this research:

- Humans' participation is justified and the benefits and risks to participants have been adequately assessed.
- A participation mechanism warranting equal opportunities for research collaboration has been developed.
- An informed consent document including the aspects required to provide the participants with the necessary information on the research has been prepared.
- It complies with the regulations in force regarding the personal data protection.

The Secretary of the SREC hereby signs this document on the date indicated below

ID. DOCUMENTO	OnxkSKUbDK		Página: 1 / 1
FIRMADO POR		FECHA FIRMA	ID. FIRMA
GONZALEZ VILLORA SIXTO		24-10-2023 10:05:25	1698135088109
OnxkSKUbDK			
